# Effect of modified air-pulse stimulation on tracheotomised patients with dysphagia after stroke: A randomized clinical trial

**DOI:** 10.1097/MD.0000000000048075

**Published:** 2026-03-13

**Authors:** Yang Gao, Wuyao Pan, Jialin Cao, Yongmei Jiang, Yunxia Du

**Affiliations:** a Department of Rehabilitation Medicine, The Second Hospital of Dalian Medical University, Shahekou District, Dalian, China.

**Keywords:** air-pulse stimulation, dysphagia, flexible endoscopy, stroke, tracheotomy

## Abstract

**Background:**

Post-stroke dysphagia is highly prevalent, particularly among severe cases requiring tracheotomy, imposing significant physical and financial burdens on patients. Currently, targeted interventions specifically designed to improve swallowing function in this tracheotomised stroke population remain limited.

**Methods:**

This study enrolled 47 subacute stroke patients with tracheotomy and dysphagia, randomized into control and trial groups. Both groups received personalized swallowing rehabilitation training, with the control group undergoing conventional air-pulse stimulation and the trial group receiving modified flexible endoscopy-mediated stimulation. Swallowing function was assessed via the Murray Secretion Scale, Penetration-Aspiration Scale, and spontaneous swallowing frequency. Additionally, the Clinical Pulmonary Infection Score assessed pneumonia severity, while hemoglobin and serum prealbumin levels evaluated nutritional status.

**Results:**

There was no statistical difference on the baseline between the 2 groups (*P* > .5). The patients in the trial group performed significantly better than the control group in all outcome indicators such as MMS (*P* < .01), Penetration-Aspiration Scale (*P* < .01), spontaneous swallowing frequency (*P* < .01), Clinical Pulmonary Infection Score (*P* < .01), hemoglobin (*P* < .05), and prealbumin (*P* < .0001), with statistical significance.

**Conclusion:**

Modified air-pulse stimulation is an effective therapy for improving swallowing function in tracheotomized stroke patients with dysphagia.

## 1. Introduction

Stroke is the second leading cause of death worldwide and a major cause of disability, which not only affects quality of life but also imposes a significant financial burden on healthcare systems.^[1]^ Dysphagia is a common symptom after stroke and occurs in up to 80% of patients in the acute phase of the disease, leading to serious complications such as malnutrition and pneumonia.^[2–4]^ Swallowing involves a group of efficient neuromuscular actions, which are regulated by a trigger center in the brainstem, namely the central pattern generator. Central pattern generator receives signal conduction from peripheral mucosal receptors, muscles and the central nervous system, thus coordinating the swallowing process.^[5,6]^ This intricate sensorimotor task involves an extensive network of cortical, subcortical, and brainstem structures. Stroke is one of the most common disorders that disrupts this swallowing network, and 11% to 50% of patients still present dysphagia after 6 months.^[7]^ If not intervened early, patients will be prone to develop permanent dysphagia. Moreover, stroke patients with dysphagia have been shown to be more likely to die than stroke patients with other complications.^[8]^

Severe stroke is clinically defined as acute stroke resulting in significant neurological impairment, typically quantified by a National Institutes of Health Stroke Scale (NIHSS) score ≥ 21. Patients with severe stroke can have different degrees of respiratory dysfunction and dysphagia for various reasons.^[9,10]^ When respiratory dysfunction is severe, airway protection is inadequate, or long-term ventilation is required, intervention by tracheotomy is required.^[11]^ Although tracheotomy may put patients out of danger in the acute phase, long-term retention of the tracheal cannula after ventilator evacuation will have a negative impact on early rehabilitation, length of stay, readmission rate, and economic costs of care.^[12]^ Tracheotomy can cause a series of physiological changes in respiratory and swallowing functions, including the inability to form normal subglottic pressure during swallowing due to reduction or disappearance of airway resistance, reduced sensitivity of the oropharynx, weak cough reflex due to decreased sensory function, reduced elevation of the larynx during swallowing, and reduced frequency of swallowing.^[13]^ These changes will to some extent exacerbate the existing swallowing dysfunction of the patient. For severe stroke patients with dysphagia, aspiration is the most fatal complication, as it can cause patients to inhale oral secretions or food residues into the lungs, leading to aspiration pneumonia. Therefore, tracheotomy is one of the most important risk factors for aspiration pneumonia in patients with dysphagia.^[14]^ Studies have reported that timely treatment of dysphagia can prevent the occurrence and development of dysphagia-related pneumonia in stroke patients.^[15–17]^ However, there is still a lack of specific targeting methods targeted at improving swallowing function in tracheotomised patients with dysphagia after stroke.

Pharyngeal hypesthesia is one of the main reasons for delayed swallowing reflex and insensitivity of cough reflex in patients.^[18]^ Therefore, restoring the sensitivity of pharyngeal receptors can improve the patient reflex arc, improve swallowing coordination, and enhance the effectiveness of the cough reflex. Current therapeutic approaches, such as air-pulse stimulation, pharyngeal electrical stimulation (PES), and speech valve therapy, rely on repeated pharyngeal mucosal stimulation to improve sensory function in tracheostomized patients with dysphagia. Building on these principles, we developed a modified air-pulse stimulation technique that combines the mechanisms of PES and speech valve therapy with the precision of flexible endoscopy. This method delivers controlled air-pulses via a humidified oxygen source connected to the endoscope, targeting swallowing dysfunction caused by reduced pharyngeal sensation. Unlike conventional air-pulse therapy that typically uses manually compressed airbags, our modified approach allows precise, repeatable oxygen pulse stimulation at specific pressures through the laryngoscope port, directly activating receptive areas associated with swallowing sensatn. Air-pulse stimulation can make air pulses work as stimulators to improve swallowing function by stimulating the oropharyngeal mucosa, inducing the swallowing reflex, and improving the sensitivity of oral and pharyngeal mucosa.^[19]^ Furthermore, sensory input can activate brain regions that correspond to the cortical swallowing network and engage both brainstem and cerebral control centers, potentially impacting the volitional components of swallowing, thereby improving swallowing function.^[20]^

This study evaluates clinical outcomes in 42 tracheotomized stroke patients receiving modified air-pulse therapy, examining its efficacy for improving swallowing function in this high-risk population. Our findings aim to establish an evidence-based approach for managing post-tracheotomy dysphagia while elucidating the therapeutic potential of sensory pathway modulation in neurogenic swallowing disorders.

## 2. Materials and methods

### 2.1. Study design

This is a prospective, randomized, parallel, controlled clinical trial. This trial enrolled 47 tracheotomised patients with dysphagia, and attempt to investigate the differences in the efficacy of modified air-pulse stimulation in the treatment of dysphagia.

**Figure 1.**
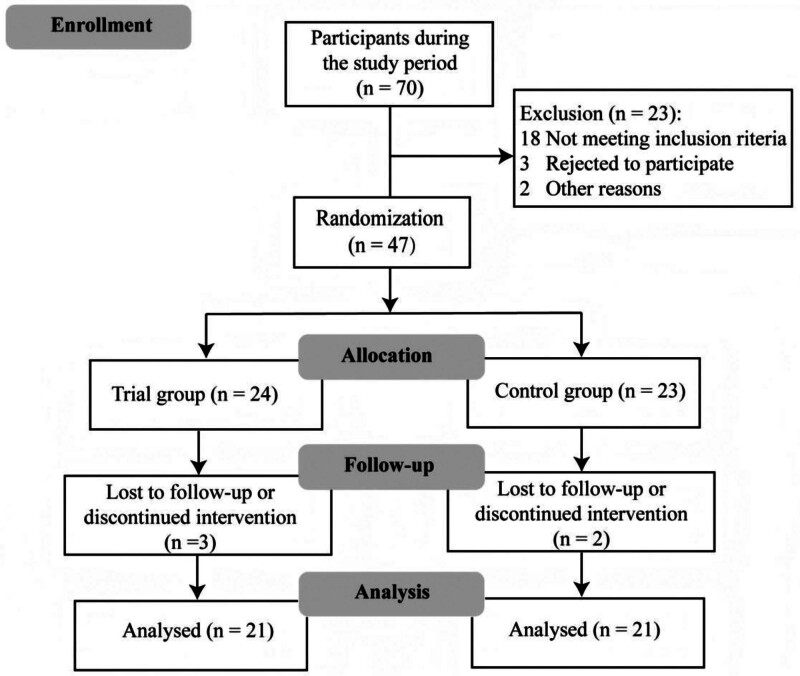
Flow diagram of participants.

We conducted this study in the rehabilitation medicine ward. Using a simple randomization method, participants (all non-mechanically ventilated) during their rehabilitation phase were randomly assigned to 2 parallel groups: the trial group (*n* = 24) and the control group (*n* = 23) through a computer-generated random binary sequence (MATLAB, MathWorks, Inc., Natick; see flow diagram in Fig. 1). Opaque, sealed envelopes numbered sequentially were used for allocation concealment. Envelopes were opened by the researcher only after participant enrollment and baseline data collection.

All participants then underwent a total of 2 weeks of swallowing function training. The trial group patients received modified air-pulse stimulation, while the control group patients received conventional air-pulse stimulation. Two designated swallowing therapists were involved in daily training, but they were not involved in any functional assessment. Additional one assessor who was not involved in the training was designated to conduct all tests to minimize the measurement bias.

This study protocol was approved by the Ethics Committee of the Second Hospital of Dalian Medical University (approval number: KY2024-048-01, approved on 05/14/2024, version number: 01). The trial was registered on ClinicalTrials.gov (registration number: NCT06299904). All participants were informed verbally and in writing about the objectives, risks, and benefits of the intervention prior to the start of the trial, and signed an informed consent form.

### 2.2. Participants

We prospectively recruited patients with dysphagia and tracheotomy after the stroke diagnosis in the Rehabilitation Department of the Second Hospital of Dalian Medical University between May 2024 and December 2024. The inclusion criteria were as follows: patients who met the diagnostic criteria for stroke formulated in the 4th National Academic Conference on Cerebrovascular disease; patients with relatively stable vital signs, with an NIHSS score of no less than 21 points; patients with tracheotomy accompanied by dysphagia; no previous history of dysphagia; age ≥ 30 years and ≤80 years; informed consent signed by the patient or their family. The exclusion criteria were as follows: patients with medullary hemorrhage/infarction; patients with sequelae of swallowing disorders caused by various diseases; patients with unstable arrhythmia, fever, infection, severe restlessness and inability to cooperate with treatment; patients with gastro-esophageal reflux, bilateral paralysis of the vocal cord, laryngopharyngeal stenosis/hemorrhage/tumor; patients in which there was an inability to accurately locate epiglottis and arytenoid cartilage due to throat disease; patients with a history of epilepsy or risk of seizures.

### 2.3. Intervention

Conventional swallowing rehabilitation training was provided comparably between the 2 groups, the control group received conventional air-pulse stimulation (5 minutes once a day for 2 weeks), while the trial group received a modified air-pulse stimulation (5 minutes once a day for 2 weeks). Conventional swallowing rehabilitation training, once a day for 30 minutes, includes: sensory comprehensive training: cold stimulation training (5 minutes), taste stimulation training (10 minutes) and vibration perception training (5 minutes); oral exercise training: muscle strength training for lips, tongue, upper and lower jaw, cheeks, and soft palate (10 minutes).

**Figure 2.**
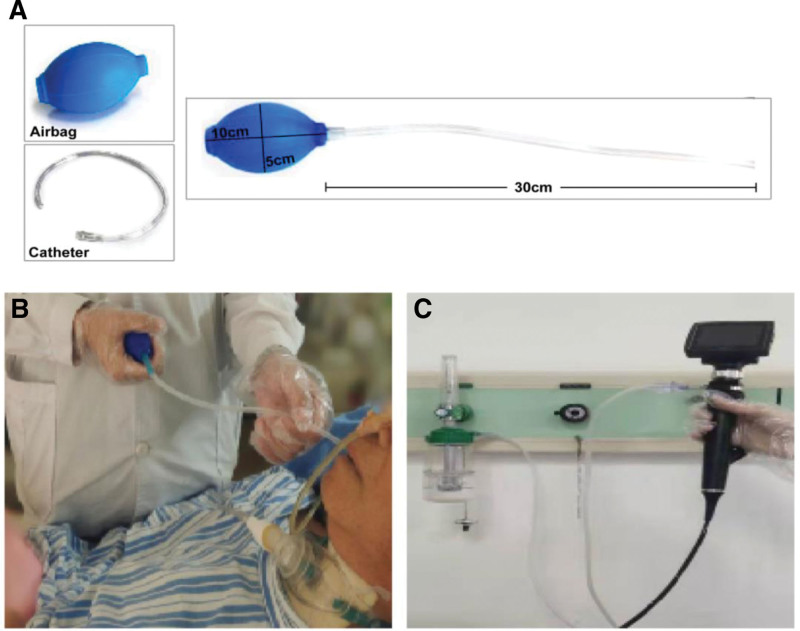
Intervention methods of 2 groups. (A) The size of airbag and catheter. (B) Conventional air-pulse stimulation treatment. (C) A flexible endoscopy was used for the modified air-pulse stimulation.

Conventional air-pulse stimulation: the terminal airway of the air pulse was placed in the oral cavity, specifically in the patient palatoglossal arch, pharyngeal posterior wall, tongue base, and other areas. The material of the catheter is silicone, with a diameter of 2 mm, and there are no side holes around the tube tip (the size of airbag and catheter are shown in Fig. 2A). The airbag was quickly squeezed to generate airflow and stimulate the mucosa (Fig. 2B). The specific operation is as follows: quickly and fully squeeze the airbag, with a frequency of 3 to 4 squeezing per second, ensuring that each area is stimulated for 3 seconds with an interval of 20 to 30 seconds.

Modified air-pulse stimulation: connected the oxygen humidification bottle to a fibreoptic endoscope, then inserted the terminal (the internal diameter of the channel is 2.2 mm) into the nasal cavity and the pulse sensation stimulatio generated by oxygen was transmitted through the internal port of the fibreoptic endoscope to the anterior wall of the pyriform recess or to the folds of the arytenoepiglottis (Fig. 2C). The treatment was performed by 2 therapists, one of whom adjusted the pressure on the wall-mounted oxygen supply to generate the air pulse. The oxygen flow volume is based on the minimum flow volume that can induce swallowing action, and the frequency and intensity of the pulses is the same as conventional air-pulse stimulation.

### 2.4. Outcome measures

#### 2.4.1. Primary outcome measures

**Figure 3.**
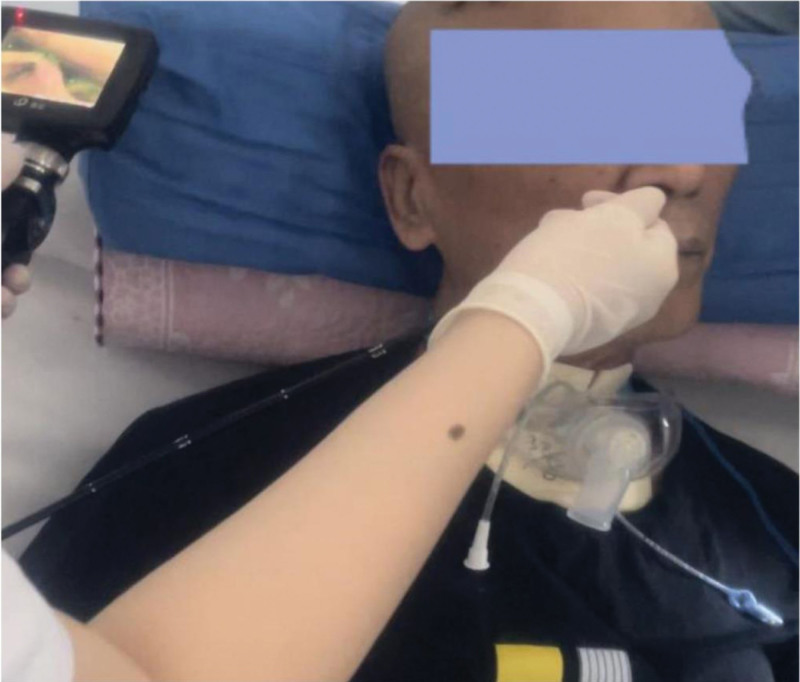
The operation of FEES. FEES = fiberoptic endoscopic examination of swallowing.

Fiberoptic endoscopic examination of swallowing was used to assess the swallowing function of all enrolled patients before and after treatment (Fig. 3), and scored with Murray Secretion Scale (MSS) and penetration-aspiration scale (PAS) according to the examination results. This operation was jointly completed by a rehabilitation physician and a swallowing therapist.

**Figure 4.**
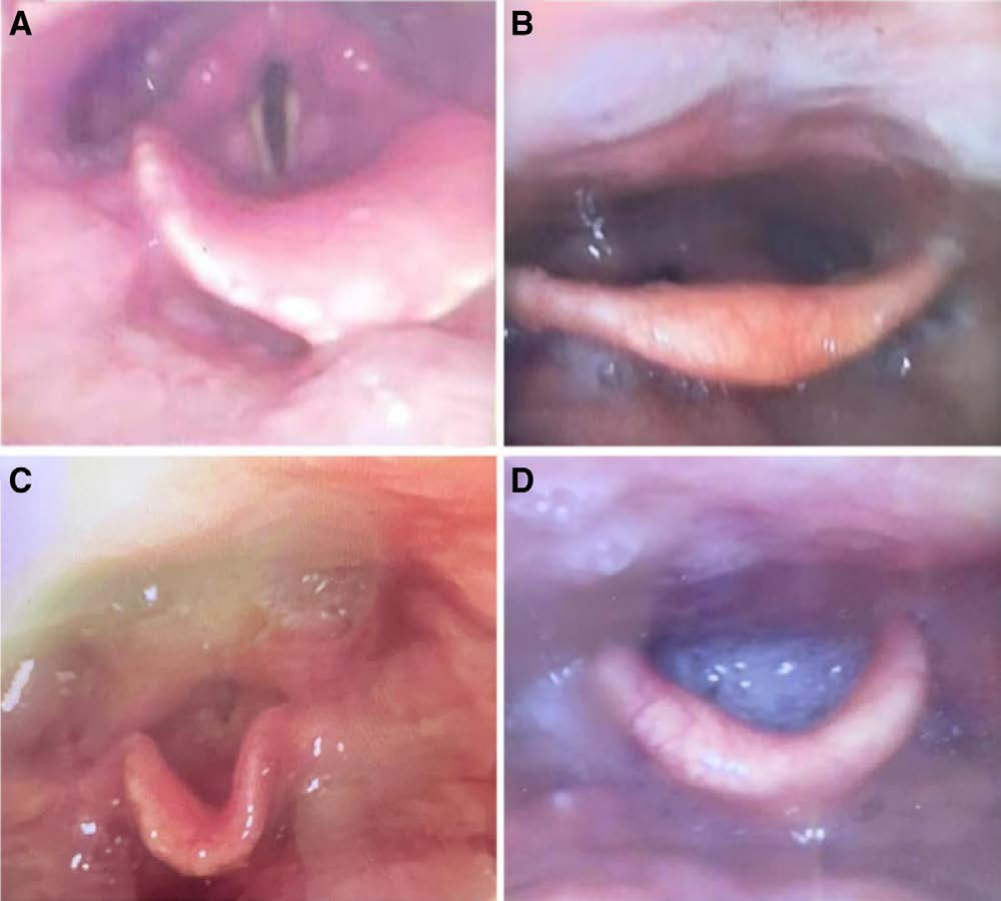
Observation of pharyngeal secretions under flexible endoscopy. (A) MSS 0: no obvious accumulation of secretion. (B) MSS 1: secretion deeply pooled in bilateral pyriform sinuses. (C) MSS 2: secretion pooled in laryngeal vestibule transiently. (D) MSS 3: secretion pooled in laryngeal vestibule consistently. MSS = Murray Secretion Scale.

The MSS is one of the validated instruments for the assessment of the accumulation of secretions in the hypopharynx, and was graded according to Table 1, based on the results of the fiberoptic endoscopic examination of swallowing. Participants without obvious saliva accumulation were assigned as grade 0 (Fig. 4A); participants with secretions pooling in the vallecula and pyriform sinus but without laryngeal penetration were assigned as grade 1 (Fig. 4B); participants who accumulated sufficient secretion during the examination, which eventually develop the presence of secretions in the laryngeal vestibule were assigned grade 2 (Fig. 4C); participants with secretions from the laryngeal vestibule at the start of the examination were assigned grade 3 (Fig. 4D).

**Table 1:** Murray Secretion Scale description.

Grade	Description
0	No visible secretions anywhere in the hypopharynx or some transient bubbles visible in the valleculae and pyriform sinuses
1	Deeply pooled bilateral secretions in the valleculae and pyriform sinuses and ending the observation segment with no visible secretions
2	Any secretions that changed from a “1” rating to a “3” rating during the observation period
3	Any secretions in laryngeal vestibule. Pulmonary secretions were included if they did not clear by swallowing or coughing

The PAS was evaluated as follows: each patient was given 5 mL of medium-thickness green-stained food to swallow, which was prepared according to the Chinese Expert Consensus on Dietary Nutrition Management for Dysphagia (2019 Edition), then swallowing function was observed under flexible endoscopy. PAS is an 8-point scale, which was designed to describe and provide a means of quantifying the severity of penetration and aspiration events during swallowing. Scores on the scale are determined primarily by the depth to which material passes in the airway and by whether or not material entering the airway is expelled: a score of 1 indicates no entry of material into the airway, scores of 2 to 5 indicate laryngeal penetration, while scores of 6 to 8 indicate tracheal aspiration (shown in Table 2).

**Table 2:** Penetration-Aspiration Scale description.

Score	Description
1	Material does not enter the airway
2	Material enters the airway, remains above vocal folds; no residue
3	Material remains above the vocal folds; visible residue remains
4	Material contacts vocal folds; no residue
5	Material contacts vocal folds; visible residue remains
6	Material passes below the glottis; no subglottic residue visible
7	Material passes below the glottis; visible subglottic residue despite patient response
8	Material passes glottis; visible subglottic residue; absent patient response

#### 2.4.2. Secondary outcome measures

For all enrolled patients, surface electromyography was used to monitor their spontaneous swallowing frequency at rest for 10 minutes before and after treatment. All enrolled patients were scored using the clinical pulmonary infection score (CPIS) based on comprehensive clinical indicators, including body temperature, leucocyte count, tracheal secretion volume and character, PaO_2_/FiO_2_ values, presence of pulmonary infiltration and microbiological culture results were recorded (shown in Table 3), to assess the patient lung infection and lung function. In addition, venous blood samples of patients were collected before and after treatment to evaluate their nutritional status, such as hemoglobin (Hb) and serum prealbumin (PAB).

**Table 3:** Clinical Pulmonary Infection Score.

Parameter	Description	Score
Temperature (°C)	≥36.5 or ≤38.4 ≥38.5 or ≤38.9 ≥39 or <36.5	0 1 2
Blood leukocytes counts (cells/mm^3^)	≥4000 or ≤11,000 <4000 or >11,000 Rod form ≥50%	0 1 2
Tracheal secretion	Tracheal secretion (−) Mild/non-purulent tracheal secretions Abundant purulent secretion	0 1 2
Oxygenation status (PaO_2_/ FiO_2_ mm Hg)	>240 or presence of ARDS ≤240 and absence of ARDS	0 2
Pulmonary infiltration in chest X-ray	No infiltration Diffuse infiltration Localized infiltration	0 1 2
Pathogenic bacteria in tracheal aspirate culture	No or few pathogenic bacteria moderate or high levels of pathogenic bacteria pathogenic bacteria to be seen in Gram staining	0 1 2

ARDS = acute respiratory distress syndrome, PaO_2_/FiO_2_ = a ratio of arterial oxygen partial pressure to fractional inspiredoxygen.

### 2.5. Sample size calculation

Sample size calculations were computed in G*Power^[21]^ using a priori power analysis. The effect size calculation was based on the best available data in the literature that aligns with the objectives and methods of the clinical trial. Using a 0.96 effect size estimation and a 2 tailed *t*-test for 2 groups, we set the desired power at 0.8 with a type-I error rate threshold of 0.05. Considering a 1:1 distribution ratio, apply the formula to calculate at least 20 participants per group. Based on previous studies, we included an additional 10% to 15% dropout rate in our calculations, resulting in an ideal sample size of 45 to 47 patients to enroll.

### 2.6. Termination criteria

The main investigator has the right to terminate the study at any time. Reasons for terminating the study include but are not limited to: continuing the study may harm the relevant rights and interests of a certain number of subjects.

### 2.7. Blindness

Collectors and evaluators will be unaware of the study protocol.

### 2.8. Statistical analysis

GraphPad Prism 9.0 software (GraphPad Software, Inc., La Jolla, CA, USA) was used for statistical analysis and graphing. Before undergoing statistical analyses, the normal distribution of data was evaluated by the Shapiro–Wilk test. Continuous variables, ordinal variables, and categorical variables were, respectively, presented as mean ± SD, medians (interquartile range, IQR), and number (percentage, %). Chi-square test and unpaired *t*-test were used to evaluate the differences between the groups in the distribution of the characteristics of the participant. For normally distributed continuous variables, an independent sample *t*-test was used for intergroup analysis, while paired sample *t*-test was used for intragroup analysis. Mann–Whitney *U* test was used for ordinal variable. Differences were considered significant when *P* < .05.

## 3. Results

### 3.1. Participant characteristics

This trial enrolled 47 patients, which were randomly assigned into the 2 parallel groups: control group (*n* = 23) and trial group (*n* = 24). General information of these 47 patients before treatment, including sex, age, type of stroke, duration of tracheotomy, NIHSS score, and medical history, were shown in Table 4. Finally, 42 patients completed the follow-up assessment and were included in the analysis. The clinical characteristics of these 42 patients were shown in Table 5. After comparing the above indicators between the trial group and the control group, it was found that there were no statistically significant differences in all data before treatment between the 2 groups of patients (*P* > .05).

**Table 4:** Baseline clinical characteristics (full analysis set, *N* = 47).

Characteristic	Control group (*n* = 23)	Trial group (*n* = 24)	*P*-value	*t* or χ2 value
Age (mean ± SD, y)	66.65 ± 9.32	64.63 ± 9.54	0.47	0.74
Sex: male (no., %)	18 (78.26)	18 (7500)	0.79	0.07
Type of stroke (hemorrhagic/ischemic)	13/10	14/10	0.90	0.02
Duration of tracheotomy (mean ± SD, d)	21.22 ± 14.37	22.71 ± 11.69	0.70	−0.39
NIHSS, mean ± SD	27.74 ± 2.70	26.92 ± 2.04	0.24	1.18
*Medical history (no., %*)				
Currently smoking	8 (34.78)	7 (29.17)	0.67	0.18
Diabetes	7 (30.43)	6 (25.00)	0.68	0.17
Hypertension	14 (60.87)	15 (62.50)	0.91	0.01
Stroke history	3 (13.04)	6 (25.00)	0.30	1.08

NIHSS = National Institutes of Health Stroke Scale.

**Table 5:** Baseline clinical characteristics (per-protocol analysis set, *N* = 42).

Characteristic	Control group (*n* = 21)	Trial group (*n* = 21)	*P*-value	*t* or χ2 value
Age (mean ± SD, y)	66.43 ± 9.74	63.52 ± 9.51	0.33	0.98
Sex: male (no., %)	17 (80.95)	16 (76.19)	0.71	0.14
Type of stroke (hemorrhagic/ischemic)	12/9	13/8	0.75	0.10
Duration of tracheotomy (mean ± SD, d)	21.10 ± 15.06	22.81 ± 12.50	0.69	−0.401
NIHSS, mean ± SD	27.81 ± 2.74	27.10 ± 1.97	0.35	0.95
*Medical history (no., %*)				
Currently smoking	7 (33.33)	6 (28.57)	0.74	0.11
Diabetes	7 (33.33)	4 (19.05)	0.29	1.11
Hypertension	13 (61.90)	15 (71.43)	0.51	0.43
Stroke history	3 (14.29)	6 (28.57)	0.26	1.27

NIHSS = National Institutes of Health Stroke Scale.

### 3.2. The results of primary outcomes

Mann–Whitney *U* tests were used for the analysis of MSS and PAS. Before treatment, there were no statistically significant differences in MSS scores between the 2 groups (*Z* = −0.305, *P* *=* .76). After treatment, the retention of secretion of patients improved in both groups compared to before treatment: control group (*Z* = −2.752, *P* = .006) and trial group (*Z* = −5.519, *P* = .0001). Compared with controls, the trial group demonstrated markedly reduced secretion post-treatment (*Z* = −3.371, *P* = .001), as shown in Table 6.

**Table 6:** MSS and PAS scores of 2 groups of patients before and after treatment.

Group (*n* = 21)	MSS		PAS	
	Before treatment	After treatment	Before treatment	After treatment
Control group median (IQR)	2 (1)	2 (1) a	7 (1)	6 (2) a
Trial group median (IQR)	3 (1)	1 (0) ab	3 (1)	1 (0) ab

MSS = Murray Secretion Scale, PAS = Penetration-Aspiration Scale.

Letter a denotes *P* < .01, *P*-values for within-group comparisons before and after treatment.

Letter b denotes *P* < .01, *P*-values for comparison between the 2 groups after treatment.

The comparison of PAS scores between the 2 groups of patients before and after treatment was shown in Table 5. Before treatment, there were no statistically significant differences in PAS scores between the 2 groups of patients (*Z* = −0.164, *P* = .87). After treatment, compared within the control group (*Z* = −3.284, *P* = .001) and within the trial group (Z = −5.123, *P* = .0001), the PAS scores of both groups of patients improved compared to before treatment. It can be seen that the improvement in the control group was relatively poor (*Z* = −3.386, *P* = .001).

### 3.3. The results of secondary outcomes

**Figure 5.**
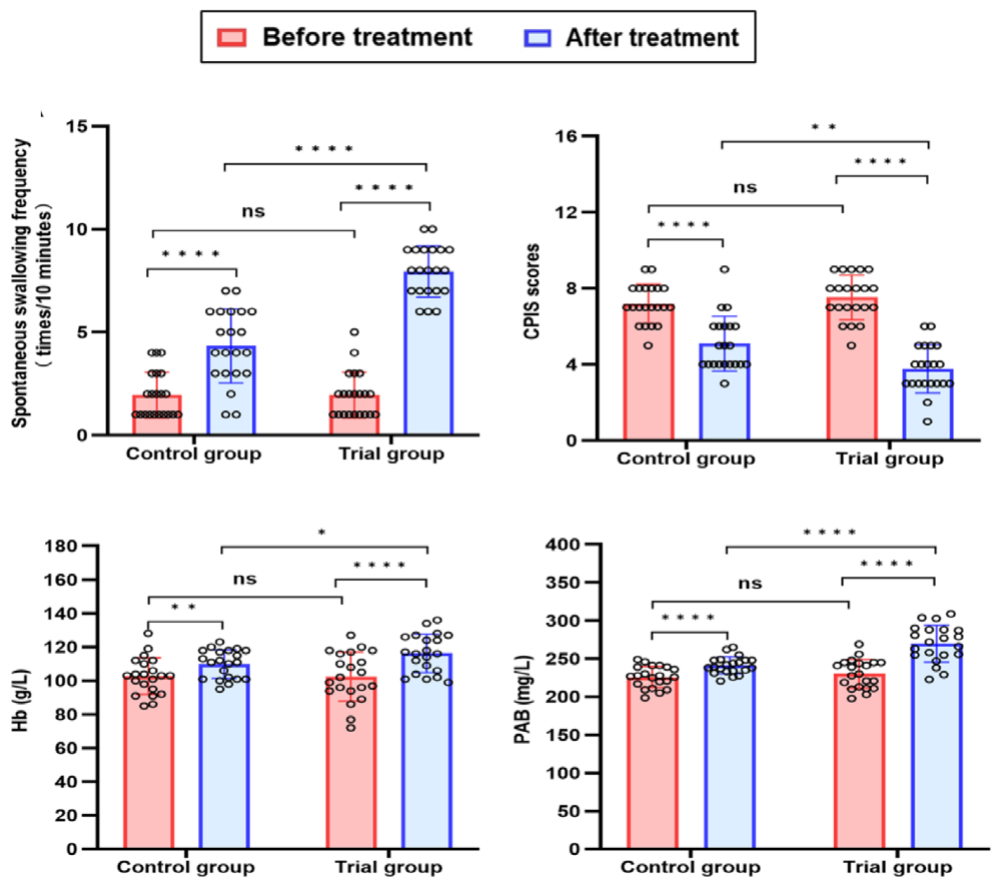
The comparison of the secondary outcomes. (A) The spontaneous swallowing frequency between the 2 groups of patients before and after treatment. (B) The comparison of CPIS scores. (C) The comparison of Hb levels. (D) The comparison of PAB levels. All data are presented as the mean ± SD. *P < .05, **P < .01, ****P < .0001. CPIS = Clinical Pulmonary Infection Score, Hb = hemoglobin, PAB = prealbumin.

As shown in Fig. 5A, there were no statistically significant differences in the spontaneous swallowing frequency between the 2 groups before treatment (*t* = 0.00, *P* > .05), 95% CI (−0.6967, 0.6967). After treatment, there was an increase in swallowing frequency both in the control group (*t* = −9.40, *P* < .0001), 95% CI (1.853, 2.909), and the trial group (*t* = −17.75, *P* < .0001), 95% CI (5.295, 6.705). The trial group demonstrated significantly improved spontaneous swallowing frequency post-treatment versus control group (*t* = −7.60, *P* < .0001), 95% CI (2.655, 4.583).

There were no statistically significant differences in CPIS scores between the 2 groups of patients before treatment (*t* = −0.98, *P* > .05), 95% CI (−0.3533, 1.020). After treatment, there was a significant decrease in CPIS both in the control group (*t* = 7.61, *P* < .001), 95% CI (−2.669, −1.521) and the trial group (*t* = 16.51, *P* < .001), 95% CI (−4.237, −3.287). In intergroup comparisons, the trial group showed a significant decrease in CPIS after treatment compared to the control group (*t* = 3.19, *P* < .01), 95% CI (−2.179, −0.4872) (Fig. 5B).

For nutritional indicators, Hb were improved compared with those before the treatment both in the control group (*t* = −3.23, *P* < .01), 95% CI (2.381, 11.05), and the trial group (Hb, *t* = −8.16, *P* < .0001), 95% CI (10.25, 17.28). Similarly, PAB were improved compared with those before the treatment both in the control group (*t* = −5.31, *P* < .0001), 95% CI (9.249, 21.23), and the trial group (*t* = −9.89, *P* < .0001), 95% CI (31.45, 48.35). In addition, the trial group showed significantly better nutritional outcomes than the control group following treatment (Hb, *t* = −2.03, *P* < .05), 95% CI (0.0386, 12.53); (PAB, *t* = −4.93, *P* < .0001), 95% CI (16.92, 40.41) (Fig. 5C and D).

### 3.4. Adverse events

Safety outcomes were assessed through systematic monitoring of adverse events. The intervention was well-tolerated, with no serious adverse events observed during the study period.

## 4. Discussion

Tracheostomy serves as a critical procedure in managing severe stroke cases, especially when brainstem injuries disrupt normal airway protection, or when damage to both sides of the brain swallowing pathways leads to chronic aspiration problems.^[22]^ The clinical determination for tracheostomy depends on multiple factors including stroke classification (hemorrhagic or ischemic), lesion localization, and institutional resource considerations.^[23,24]^ In this study, although baseline characteristics showed no statistically significant differences between groups, including sex, age, stroke type, duration of tracheotomy, NIHSS score, and medical history, we observed an interesting divergence in stroke type distribution from patterns reported in prior literature. A meta-analysis included 17,346 tracheostomized patients from 13 studies, revealing a notable discrepancy in stroke subtype distribution compared to our study. While ischemic stroke represented only 12% of cases in the aggregated studies, our study population exhibited a markedly higher proportion of ischemic stroke patients (42.6%).^[24]^ However, this finding aligns with a retrospective Chinese study of 61 stroke patients undergoing tracheotomy, which reported comparable proportions (39.3% ischemic versus 60.7% hemorrhagic),^[25]^ supporting the validity of our observations. This discrepancy reflects our center unique patient population resulting from structural limitations in admitting severe hemorrhagic stroke cases. As a branch with neurology but no neurosurgery, our center faces logistical challenges in transferring patients with severe hemorrhagic stroke. Therefore, compared to ischemic stroke patients, our center admits much fewer cases of hemorrhagic stroke. These structural factors should be considered when interpreting tracheostomy outcomes in stroke rehabilitation studies.

Tracheostomy-associated dysphagia constitutes a serious medical complication in stroke patients, carrying significant risks of aspiration and malnutrition.^[26]^ Our findings establish that modified air-pulse therapy significantly enhances swallowing recovery in tracheotomized stroke patients, offering a promising solution to this high-risk population dysphagia challenges. Our novel endoscopy-guided oxygen pulse therapy enables targeted pharyngeal stimulation, demonstrating excellent clinical efficacy in secretion control (assessed by MSS grade) and prevention of aspiration (assessed by PAS score), with an 83.6% increase in spontaneous swallowing frequency, and a 26.3% decrease in CPIS score. These findings support Heidler MD hypothesis that natural airflow plays a critical role in eliciting swallowing reflexes and highlight the importance of sensory feedback restoration in dysphagia management.^[27]^ Moreover, the modified air-pulse therapy group showed improvements in nutritional indicators compared to controls, with Hb increased by 5.7% and PAB increased by 11.9%. These nutritional benefits likely resulted from the therapy dual effects on reducing aspiration-related pulmonary infections and systemic inflammation while simultaneously decreasing metabolic demands. By improving swallowing function, the intervention created a favorable metabolic environment that enhanced protein synthesis and reduced catabolic losses, even before patients resumed oral feeding.

### 4.1. Neural plasticity and sensory restoration mechanisms

Pharyngeal hypesthesia has been reported to be an independent predictor of the severity of post-stroke dysphagia and impaired secretion management.^[28]^ Long-term tracheotomy or mechanical ventilation can cause patients to experience changes in the mechanical and chemical receptors of the pharyngeal and laryngeal mucosa. These alterations are due to lack of physiological airflow through the pharynx and larynx, as well as muscle atrophy and gradual loss of proprioception, resulting in reduced or lost swallowing and cough reflexes, gastro-esophageal reflux, and respiratory swallowing asynchrony.^[26]^ These factors increase the probability of aspiration, especially implicit aspiration.

The neural repair mechanism and increased cortical activity play a crucial role in recovery from stroke, and the central nervous system exhibits plasticity and reorganization ability in both structure and function.^[29]^ The increased sensory input of the pharynx is beneficial in enhancing the ability to drive swallowing cortical control and reorganization related to swallowing cortical function.^[30]^ Studies have shown that stimulating the sensory area innervated by the pharyngeal plexus formed by the pharyngeal branch of the vagus nerve and the glossopharyngeal nerve is effective in triggering or regulating the swallowing reflex, as well as eliciting the defensive cough reflex and the laryngeal adduction reflex.^[31]^ These sensory areas correspond to the laryngeal mucosa in the epiglottis and arytenoid cartilage, the pharyngeal sidewall, and the surrounding tonsils. Previous studies have shown that repeated sensation stimulation is beneficial for the formation of functional neural pathways, consolidating the efficiency of synaptic creation or activation, activating relevant functional areas, and promoting cortical reorganization to accelerate the recovery process.^[32]^

### 4.2. Advantages over existing interventions

PES is a therapeutic intervention involving the delivery of controlled electrical currents to the pharyngeal mucosa via a transnasally inserted catheter.^[33]^ Compared with electrical stimulation, air-pulse stimulation stimulates the pharyngeal mucosa through airflow pressure and induces swallowing reflex, increasing the sensitivity of the pharyngeal mucosa and improving the speed of swallowing initiation, rarely causes discomfort in patients and does not cause aspiration, which makes it safer.^[34]^ It is particularly suitable for patients with severe cognitive impairment and inability to cooperate with other swallowing exercises.

The speech valve is a very small and portable one-way valve that can form a closed path for the respiratory tract.^[35]^ The use of a speech valve in tracheostomized patients has been shown to restore normal subglottic air pressures, a key component of swallowing, thus potentially improving swallow function and reducing the risk of aspiration.^[36]^ The similarity between modified air-pulse stimulation and the speech valve is that both can provide airflow stimulation to the patient throat to improve swallowing sensitivity, but the speech valve is suitable for conscious patients who can spontaneously expel phlegm and have no risk of aspiration. Patients with severe conditions and prolonged tracheotomy may experience disuse atrophy of their respiratory muscles and have a weak cough reflex, which results in the inability to expel phlegm from their mouths, which can lead to re-inhalation of phlegm into the lungs and cause infection. Second, when patients have decreased respiratory function, they cannot tolerate the use of a speech valve, and the speech valve cannot be used in patients with consciousness disorders.^[37]^

At present, the devices used for conventional air-pulse stimulation are mostly simple airbags connected to the catheter.^[38]^ The air pulse generated by manually pressing the airbag has problems such as unstable stimulation pressure, inaccurate positioning, and insufficient accuracy. While the modified air-pulse stimulation can accurately and repeatedly stimulate the swallowing sensation related sensory area with oxygen pulses through the laryngoscope port under specific pressure. Repeated pressure stimulation can increase the sensitivity of receptors and related nerve endings, thereby promoting the recovery of sensory feedback in the patient throat, inducing the laryngeal adduction reflex, and enhancing the patient airway protection ability. Moreover, it can increase the patient autonomous swallowing frequency, promote the recovery of muscle strength and coordination related to swallowing, and ultimately improve swallowing function.

### 4.3. Clinical implications

This study demonstrates that our innovative air-pulse therapy effectively improves swallowing function in post-tracheotomy stroke patients with dysphagia. By combining controlled air-pulse stimulation with endoscopic visualization, the technique successfully compensates for upper airway sensory deprivation. The observed improvements in swallowing frequency, secretion control, and reduced aspiration risk support its clinical potential. While our findings specifically apply to adult stroke populations, we recognize pediatric stroke, although less common, represents a significant cause of childhood mortality and neurological disability,^[39]^ where dysphagia management presents unique challenges. Future research should investigate tracheostomy-related outcomes in pediatric populations to establish evidence-based, age-specific protocols. Furthermore, this method is worth evaluating in some neurodegenerative diseases that share common risk factors and pathological mechanisms with stroke,^[40]^ which may expand its clinical applicability.

### 4.4. Limitations

This study has some limitations. Firstly, this is a pilot study, our main objective is to collect preliminary data and evaluate the feasibility of the intervention, so the sample size of our study is relatively small. While we conducted a power analysis to inform the sample size, we acknowledge that the limited number of participants may affect the generalizability of the effectiveness results and the ability to detect rare adverse events. In addition, the use of videofluoroscopic swallow study (VFSS) is the gold standard for diagnosing swallowing disorders. However, this study did not use VFSS to evaluate swallowing function due to budget constraints. Lastly, we just explored the improvement of swallowing function by modified air-pulse stimulation, without measuring the changes in swallowing activity or sensory function in the central cortex through fMRI or functional near-infrared spectroscopy, to elucidate its possible mechanisms. In future studies, we will further explore its mechanism to obtain more conclusive results.

## 5. Conclusion

In recent years, the rehabilitation of stroke patients with tracheotomy has received increasing attention, but there are relatively few methods to accelerate the improvement in swallowing in patients with tracheotomy. In this clinical study, we have studied a treatment method that can improve swallowing disorders in stroke patients with tracheotomy. The results indicate that, compared to traditional treatment methods, modified air-pulse stimulation can improve swallowing disorders and secretion management skills in patients, leading to a reduction in lung infection rates. This provides a new treatment strategy for swallowing function in stroke patients with tracheotomy combined with dysphagia, which deserves clinical promotion and application.

## Acknowledgments

We are indebted to the subjects who participated in the study for their consent and cooperation. Meanwhile, we acknowledge the contributions of the Department of Rehabilitation Medicine, the Second Hospital of Dalian Medical University that aided the efforts of the authors.

## Author contributions

**Data curation:** Yang Gao, Wuyao Pan.

**Investigation:** Yang Gao, Wuyao Pan.

**Methodology:** Yang Gao, Wuyao Pan.

**Resources:** Yang Gao, Wuyao Pan.

**Writing – original draft:** Yang Gao.

**Formal analysis:** Jialin Cao.

**Software:** Jialin Cao.

**Conceptualization:** Yongmei Jiang, Yunxia Du.

**Supervision:** Yongmei Jiang.

**Validation:** Yongmei Jiang, Yunxia Du.

**Project administration:** Yunxia Du.

**Writing – review & editing:** Yunxia Du.
